# A Microsomal Proteomics View of H_2_O_2_- and ABA-Dependent Responses

**DOI:** 10.3390/proteomes5030022

**Published:** 2017-08-18

**Authors:** May Alqurashi, Ludivine Thomas, Chris Gehring, Claudius Marondedze

**Affiliations:** 1 Cambridge Centre for Proteomics, Cambridge Systems Biology Centre, Department of Biochemistry, University of Cambridge Tennis Court Road, Cambridge CB2 1QR, UK; may.qurashi@kaust.edu.sa; 2 Biological and Environmental Sciences & Engineering Division, King Abdullah University of Science and Technology, Thuwal 23955-6900, Saudi Arabia; c.a.gehring@molecular-signals.com; 3 HM. Clause, rue Louis Saillant, Z.I. La Motte, BP83, 26802 Portes-lès-Valence, France; ludivinet1@gmail.com; 4 Department of Chemistry, Biology & Biotechnology, University of Perugia, Borgo XX giugno 74, 06121 Perugia, Italy; 5 Laboratoire de Physiologie Cellulaire et Végétale, Université Grenoble Alpes, CEA/BIG, 17, avenue des Martyrs, 38054 Grenoble, France

**Keywords:** hydrogen peroxide (H_2_O_2_), abscisic acid (ABA), microsomal proteomics, quantitative proteomics, mass spectrometry

## Abstract

The plant hormone abscisic acid (ABA) modulates a number of plant developmental processes and responses to stress. In planta, ABA has been shown to induce reactive oxygen species (ROS) production through the action of plasma membrane-associated nicotinamide adenine dinucleotide phosphate (NADPH)-oxidases. Although quantitative proteomics studies have been performed to identify ABA- or hydrogen peroxide (H_2_O_2_)-dependent proteins, little is known about the ABA- and H_2_O_2_-dependent microsomal proteome changes. Here, we examined the effect of 50 µM of either H_2_O_2_ or ABA on the Arabidopsis microsomal proteome using tandem mass spectrometry and identified 86 specifically H_2_O_2_-dependent, and 52 specifically ABA-dependent proteins that are differentially expressed. We observed differential accumulation of proteins involved in the tricarboxylic acid (TCA) cycle notably in response to H_2_O_2_. Of these, aconitase 3 responded to both H_2_O_2_ and ABA. Additionally, over 30 proteins linked to RNA biology responded significantly to both treatments. Gene ontology categories such as ‘response to stress’ and ‘transport’ were enriched, suggesting that H_2_O_2_ or ABA directly and/or indirectly cause complex and partly overlapping cellular responses. Data are available via ProteomeXchange with identifier PXD006513.

## 1. Introduction

Plants, much like animals, are susceptible to oxidative damage by reactive oxygen species (ROS). The production of ROS, and in particular hydrogen peroxide (H_2_O_2_), increases during exposure to abiotic stress [1,2,3] and pathogen infection [4]. In pathogen infections, suppression of ascorbate peroxidase and catalase during the hypersensitive response enhances pathogen-induced programmed cell death [4]. The mitochondrial electron transport chain also synthesises a significant amount of ROS, mainly in the form of superoxide [5] that can cause oxidative stress [6,7]. Previous studies have shown that the oxidative stress imposed by H_2_O_2_ is a potent inhibitor of tricarboxylic acid (TCA) cycle enzymes such as citrate synthase [8], aconitase, and succinyl CoA ligase [9,10,11]. However, at low concentrations, H_2_O_2_ functions as a stress signal [12,13].

It has been demonstrated that ROS production is required for abscisic acid (ABA) signal transduction in guard cells [14,15]. In the guard cells, ABA is perceived by PYRABACTIN resistance (PYR)/PYR-Like 1/Regulatory components of ABA receptors (RCAR) [16,17], which in turn induce the production of ROS including H_2_O_2_, by nicotinamide adenine dinucleotide phosphate (NADPH) -oxidase [18,19]. ABA also induces stomatal closure but interestingly, treating the guard cells of ABA-insensitive mutants, *abi1-1*, with ABA did not induce ROS production but activation of hyperpolarised-activated Ca^2+^ (*I*_Ca_) channels and the induction of stomatal closure by H_2_O_2_, suggesting that *abi1-1* disrupts ABA signalling between ABA reception and ROS production [20]. Besides, ABA regulates many plant developmental processes and induces increased tolerance to different stresses such as drought, salinity and low temperature [21]. ABA-induced ROS in the mitochondria of root tip cells operates as retrograde signal that regulate meristem activity in Arabidopsis [22]. In maize (*Zea mays*) leaves, water stress-induced ABA accumulation has been detected to elicit an increased production of H_2_O_2_ [23,24]. Also, in rice (*Oryza sativa*) seedlings, exogenous ABA has been observed to increase H_2_O_2_ content in leaves grown under potassium sufficient conditions [25].

Despite the considerable body of information on role of ABA in stress responses, the post-translational molecular targets at the microsomal level and particularly their relation with H_2_O_2_ is yet to be properly understood. Therefore, we set to find out whether H_2_O_2_ and ABA can induce a common response at the microsomal proteome level. To do this, downstream microsomal protein changes were investigated using label-free quantitative mass spectrometry analysis. Further, to find out whether ABA induces H_2_O_2_ production or *vice versa*, we tested for H_2_O_2_ production upon ABA treatment of *Arabidopsis thaliana* ecotype Columbia-0 cell suspension cultures or ABA production upon H_2_O_2_ treatment. In this study we showed that H_2_O_2_ and ABA have common and independent responses. The former is however, not surprising, as ABA has previously been shown to induce production of H_2_O_2_, thus the common response suggests an H_2_O_2_-dependent response, which can also be elicited via ABA.

## 2. Materials and Methods

### 2.1. Treatments of Arabidopsis Cell Suspension Culture

Cells derived from *Arabidopsis thaliana* (ecotype Columbia-0) roots were grown in Gamborg’s B5 basal salt mixture (Sigma-Aldrich, St. Louis, MO, USA), as described in [26,27]. At Day 7 post-subculturing, three biological replicate flasks containing cells were treated with 50 μM ABA or 50 μM H_2_O_2_ for 0 min (mock treatment), 5 min and 20 min. Cells were then harvested by draining off the media using a Stericup^®^ filter unit (Millipore, Billerica, MA, USA), immediately flash frozen in liquid nitrogen, and stored at −140 °C until further use.

### 2.2. Microsomal Protein Isolation

Approximately 1 g of cells was ground to a fine powder in liquid nitrogen and subjected to microsomal isolation, as described in [28]. The powder was incubated in a sucrose buffer (50 mM Tris(hydroxymethyl)aminomethane (pH 8.0), 2 mM ethylenediaminetetraacetic acid (EDTA), 2 mM dithiothreitol (DTT), 0.25 M sucrose and 1× protease inhibitor cocktail tablet (Sigma-Aldrich, St. Louis, MO, USA)) and centrifuged at 8000× *g* for 15 min. The supernatant was subjected to ultracentrifugation using an Optima™ L-100K ultracentrifuge (Beckman Coulter, Brea, CA, USA) at 100,000× *g* for 1 h. The supernatant, representing the cytosolic fraction, was pipetted out. The pellet, the microsomal fraction, at the bottom of the tube, was washed once in sucrose buffer and centrifuged at 100,000× *g* for 1 h. The microsomal fraction corresponding to the final pellet was suspended in sucrose buffer, aliquoted into separate tubes and either used immediately or stored at −80 °C.

### 2.3. Trypsin Digestion and Protein Identification by Tandem Mass Spectrometry

Approximately 0.2 mg of total microsomal protein extract was reduced with 5 mM DTT for 2 h at 37 °C and cooled. The sample was then alkylated with 14 mM iodoacetamide for 30 min at room temperature in the dark. Unreacted iodoacetamide was quenched by increasing DTT concentration to 10 mM and incubated for 15 min at room temperature in the dark. Proteins were incubated at 50:1 ratio with sequencing-grade modified trypsin (Promega, Madison, WI, USA) overnight at 37 °C with gentle agitation. Protein digestion was stopped by acidification of the mixture to pH 2.0 with trichloroacetic acid. Peptides were desalted using Sep-Pak Vac tC18 100 mg cartridges (Waters, Milford, MA), as described previously [27]. After desalting, peptides were re-suspended in 5% (*v*/*v*) acetonitrile and 0.1% (*v*/*v*) formic acid and analysed by the LTQ-Orbitrap Velos mass spectrometer (Thermo-Scientific, Bremen, Germany) coupled with a nanoelectrospray ion source (Proxeon Biosystems, Odense, Denmark) for nano-liquid chromatography tandem mass spectrometry (LC-MS/MS) analyses as described in [29]. Five microlitres of the peptide mixtures was injected onto a 50 mm long × 0.3 mm Magic C18AQ (Michrom) column. A spray voltage of 1500 V was applied and the MS scan range used was *m/z* 350 to 1600. The top 10 precursor ions were selected in the MS scan by the Orbitrap with a resolution r = 60,000 for fragmentation in the linear ion trap using collision-induced dissociation. Normalised collision-induced dissociation was set at 35.0 and spectra were submitted to a local MASCOT (Matrix Science, London, UK) server and searched against Arabidopsis in the Arabidopsis Information Resource (TAIR, Release 10), with a precursor mass tolerance of 10 ppm, a fragment ion mass tolerance of 0.6 Da, and strict trypsin specificity allowing up to one missed cleavage. Carbamidomethyl modification on cysteine residues was selected as fixed modification, and oxidation of methionine residues as variable modification. Identified proteins were further validated with Scaffold version 4.0.4 (Proteome Software, Portland, OR, USA). Identified proteins were considered positive with a molecular weight search (MOWSE) score ≥32, number of peptides ≥2, a 95% protein and peptide probability, and a false discovery rate (FDR) ≤1%. Label-free quantitative analysis was performed using spectral counts with Scaffold software. Proteins were deemed responsive to treatment when present in at least two replicates with a fold change (FC) greater or equal to |±2|, verified by Student t-test (*p*-value ≤ 0.05) in comparison to the mock-treated samples. All proteomics methods are extensively detailed elsewhere [26,29,30,31].

### 2.4. Intracellular ROS Assay and ABA Measurements

Cellular ROS levels were measured using OxiSelect™ intracellular ROS assay kit green fluorescence (Cambridge Bioscience, Cambridge, UK) after 20 min of 50 μM ABA treatment. The procedure used was according to the manufacturer’s protocol. ABA contents in samples treated with 50 μM H_2_O_2_ for 5 and 20 min were measured using Phytodetek^®^ immunoassay kit for ABA (Agdia, Elkhart, IN, USA) according to the manufacturer’s instructions.

### 2.5. Bioinformatic Analyses

The gene ontology (GO) analysis toolkit in the database AgriGO [32] was used for the detection of enriched cellular components, biological processes and molecular functions.

## 3. Results

To gain insight on the microsomal cellular responses to H_2_O_2_ and ABA, a comparative proteomic analysis was undertaken. Microsomal proteins were isolated from Arabidopsis cell suspension cultures pre-treated with 50 µM of either H_2_O_2_ or ABA for 5 min or 20 min, digested with trypsin and analysed by MS/MS. Data was processed with MASCOT and Scaffold for identification and label-free quantitation. A total of 906 proteins from the enriched samples were identified (Table S1). Of these, 86 proteins were significantly (*p* ≤ 0.05) responsive to H_2_O_2_ treatment (Table 1) and 52 proteins to ABA treatment (Table 2). A total of 21 responsive proteins were common to both ABA and H_2_O_2_ treatments and majority of the proteins displayed similar response signatures (Table 3).

### 3.1. H_2_O_2_-Responsive Microsomal Proteins

Of the 86 H_2_O_2_-dependent significantly changing proteins, 24 proteins increased and 42 proteins decreased in abundance at 5 min, while, 38 proteins increased and 18 proteins decreased in abundance at 20 min. These proteins were classified into 12 major functional categories [31] (Table 1, in depth detail in Table S2). The most represented functional categories include transporters (17%), energy (15%) and protein synthesis (14%) (Table 1). Gene ontology (GO) analysis of the H_2_O_2_ responsive proteins revealed enrichment in biological processes such as ‘cellular process’, ‘metabolic process’, ‘response to stress’ and ‘transport’ (Table S2). In addition, enriched molecular function categories included ‘structural molecule activity’, ‘transporter activity’ and ‘oxidoreductase activity’ (Table S2).

**Figure 1:**
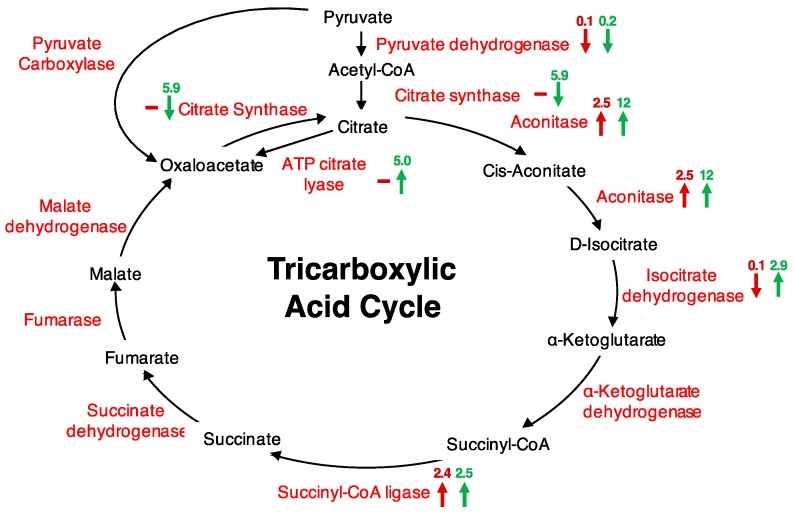
Tricarboxylic acid (TCA) cycle highlighted proteins changing in abundance after H_2_O_2_ treatment of cells. Maroon arrows represent proteins changing at 5 min after treatment and green arrows at 20 min after treatment. “−” signifies no change or a change of less than two-fold, “↑” signifies an increase in abundance, and “↓” signifies a decrease in abundance. Respective protein fold changes are indicated on top of each arrow.

Further, we performed metabolic pathway analyses using the Kyoto Encyclopedia of Genes and Genomes (KEGG) database to find out if any pathways were represented. The ‘purine metabolism’ (13 proteins), ‘thiamine biosynthesis’ (11 proteins), and ‘citrate cycle (TCA cycle)’ (six proteins) (Table S2) were the most represented. The abundance of five of the six proteins associated with the TCA cycle increased, particularly at 20 min after H_2_O_2_ treatment (Figure 1). The responsive proteins included pyruvate dehydrogenase (AT1G24180), an essential precursor that links glycolysis to the TCA cycle, citrate synthase (AT2G44350), aconitase 3 (AT2G05710), isocitrate dehydrogenase (AT1G65930), succinyl-CoA ligase, alpha subunit (AT5G08300), and ATP citrate lyase/succinyl-CoA synthetase (AT2G20420), an enzyme that cleaves citrate to oxaloacetate and acetyl CoA in the presence of ATP and CoA, and is implicated in carbohydrate metabolism and production of fatty acids (Table 1; Figure 1). Citrate synthase catalyses the first committed step in the TCA cycle, and has been shown to decrease in activity by 54% upon oxidation by H_2_O_2_ [8]. Site-directed mutagenesis of six cysteine residues of citrate synthase showed that the mutant proteins could convert acetyl CoA to oxaloacetate with decreased efficiencies compared to the unmodified citrate synthase. The Cys108Ser and Cys325Ser had their activities decreased by 98%, suggesting that the Cys108 and Cys325 are important for citrate synthase activity. These two nearly inactive forms showed high insensitivity to H_2_O_2_, while other mutants just like the unmodified citrate synthase had decreased activities upon H_2_O_2_ treatment [8]. Similar to the observed change in citrate synthase upon H_2_O_2_ treatment [8], we observed citrate synthase together with pyruvate dehydrogenase significantly decreasing in abundance, particularly 20 min after treatment. Furthermore, it has been shown that pyruvate dehydrogenase and the TCA enzymes, including citrate synthase and aconitase, are sensitive to H_2_O_2_ [9]. Of these, aconitase is the most sensitive to the H_2_O_2_ effect [9]. Aconitase is one of the five enzymes that exerted the most influence on controlling the TCA, with a control coefficient of 0.964 (the control coefficient is the measure of the ratio of relative change in flux and the relative change in enzyme amount [33]), the second highest after malate dehydrogenase (1.76) [34]. Other enzymes identified that changed in response to H_2_O_2_ had a lower control coefficient; for example citrate synthase −0.4, isocitrate dehydrogenase −0.123 and succinyl CoA ligase 0.0008 [34]. Aconitase converts citrate to isocitrate and contains a Fe-S cluster that is one of the primary target sites for ROS effect [35]. In animals, aconitase activity is suppressed by treatment with 50 μM H_2_O_2_, that in turn causes reduced TCA cycle activity in the cardiac cells [36]. In plants, aconitase affects superoxide dismutase transcription, and has been implicated in regulating resistance to oxidative stress, cell death and salt stress in Arabidopsis and *Nicotiana benthamiana* [37]. This phenomenal role of aconitase in regulating oxidative stress was also supported by our data, in which the abundance of aconitase increased in response to H_2_O_2_ as well as ABA (Table 2). The latter may have been an ABA-dependent response or potentially an ABA-dependent H_2_O_2_ response. Aconitase has also been observed to increase in abundance in response to cyclic guanosine 3′, 5′-monophosphate (cGMP), a second messenger that also showed a time- and concentration-dependent ROS production [28]. It is important to note that the observed increase in aconitase 3 did not necessarily reflect an increase in its activity that consequently could lead to a TCA flux. This is so because it has been shown that aconitase activity is strongly reduced by H_2_O_2_ [9,10,11]. Rather, the increase may have been channelled towards its role as RNA-binding protein, where it regulates transcription and stability of mRNA transcripts, including its own [38,39]. Besides, the role of aconitase in RNA-binding and oxidative stress requires further analysis. Overall, a total of 36 H_2_O_2_—responsive proteins were experimentally linked to potential RNA interaction, including the other TCA cycle enzymes, isocitrate dehydrogenase, succinyl CoA ligase, and succinyl CoA synthase, that increased in abundance at 20 min post H_2_O_2_ treatment [39]. Taken together, an increase in the aconitase in response to ABA, cGMP or H_2_O_2_ may suggest a potential role of these signalling molecules in modulating oxidative stress as well as energy supplies.

### 3.2. ABA-Responsive Microsomal Proteins

A total of 52 proteins showed differential accumulation in response to ABA and 26 of these proteins have been identified as either RNA-binding proteins or candidate RNA interactors [39]. Abundance of 31 proteins increased and 18 decreased at 5 min after treatment with ABA, while at 20 min, 28 proteins increased and 17 proteins decreased (Table 2). The 52 proteins were classified into 11 functional categories [31], and the most represented functional categories were protein synthesis (19%), metabolism (13%), cell structure (13%), and energy (12%) (Table 2; extended summary in Table S3). A functional enrichment analysis of the ABA-responsive proteins revealed significant enrichment of biological processes, including ‘primary metabolic process’, ‘developmental process’, ‘response to stress’, ‘transport’, and ‘translation’ (Table S3). In addition, the molecular functions were also enriched, and these included ‘structural molecule activity’ and ‘transporter activity’ (Table S3). A metabolic pathway analysis was also performed on the ABA-responsive proteins using KEGG, and ‘purine metabolism’ (seven proteins), ‘thiamine metabolism’ (six proteins), and ‘methane metabolism’ (four proteins) were the most represented pathways (Table S3).

Fifteen proteins decreased in abundance at both 5 and 20 min after ABA treatment, and these included six cytoskeleton-associated proteins (Table 2). Three of these were transporter-associated proteins namely clathrin, heavy chains (AT3G08530 and AT3G11130) and a plasma membrane intrinsic protein 2A (PIP2A, AT3G53420). The latter has been linked to other stress-responses, including temperature and salt [40,41], in addition to a potential role in mRNA binding [39]. The abundance of PIP2A, in particular, has been shown to decrease in response to salt stress [41,42], and much like in the current study, the abundance of PIP2A decreased after 5 and 20 min (although at 20 min it was only 0.6 fold change, and hence less than the cutoff of two-fold) of ABA-treatment. PIP2A is a member of the PIP2 subfamily proteins. PIP2A has water transport activity as depicted in *Xenopus laevis* oocytes [43], and facilitates the diffusion of H_2_O_2_ into cells of yeast [44]. Exposing of Arabidopsis roots to 0.5 mM H_2_O_2_ has been observed to induce significant depletion of PIP homologs in the plasma membrane fractions after 15 min. In addition, in late endosomal compartments, H_2_O_2_ has been shown to cause oxidative stress-induced redistribution of *At*PIP2.1 [45]. On another hand, an ABA-dependent phosphoproteomics analysis detected four members of the aquaporin family including PIP2 decreasing in phosphorylation state at a carboxyl-terminal serine that is anticipated to instigate closure of the water-transporting aquaporin gate [46]. PIP2 dephosphorylation prevents rehydration during ABA-regulated seed germination and dormancy, and decreases water flux in response to drought [46]. Arabidopsis knockout mutants lacking the PIP2:1 aquaporin have a stomatal closure defect particularly in response to ABA [47]. In *pip2:1* plants, ABA treatment induced an increase in osmotic water permeability of guard cell protoplasts while abolishing accumulation of ROS [47], supporting the involvement of aquaporins in ABA-dependent stomatal movements. The work by Grondin et al*.* [47] implies that PIP2:1 does significantly contribute to guard cell water permeability in the presence of ABA. Moreover, just like Kline et al*.* [46], Grondin et al*.* [47] showed that PIP2 phosphorylation is critical for responses to ABA, in particular in guard cell movements. In the current study, differential analysis of microsomal proteins showed that accumulation of PIP2 decreased in the presence of ABA, suggesting a potential direct or indirect regulation of aquaporins in ABA signalling. This may correlate with the decrease in phosphorylation of PIP2 upon ABA treatment [46].

Among the microsomal proteins that increased in abundance after ABA treatment, D-mannose-binding lectin protein (AT1G78850) and LRR transmembrane protein kinase (LTPK, AT3G14840) (Table 2) showed the greatest increase specifically at 20 min. The two proteins play a role in inmate immunity against microbial attack. D-mannose-binding lectin recognises certain carbohydrate moieties on pathogen surfaces [48]. It is involved in the regulation of gene expression and cellular activities in response to increasing levels of hormones such as ABA and jasmonate, or biotic and abiotic (e.g., osmotic) stresses [49,50,51,52,53]. On the other hand, LTPK [also known as LYSM RLK1-INTERACTING KINASE 1 (LIK1)], a plasma membrane localised protein, also increased in abundance at 20 min after H_2_O_2_ treatment. Direct interaction between chitin elicitor receptor kinase (AtCERK1) and LIK1, both in vitro and in vivo studies showed that LIK1 is directly phosphorylated by AtCERK1 [54]. In Arabidopsis, *AtCERK1* mutants show impairment in chitin responses including the activation of a MAPK cascade, ROS production and expression of chitin-induced genes consequently leading to failure of chitin-induced pathogen resistance [55,56]. Furthermore, *Lik1* T-DNA mutant plants produced significantly more ROS in response to chitin and showed an increased resistance to *Pseudomonas syringae* pv. *tomato* [54]. The increase in abundance of LTPK in response to the ABA and H_2_O_2_ treatments we observed was consistent with the role of LTPK in ROS responses for regulating ROS accumulation that, if in excess, can facilitate necrotrophic infection and promotes programmed cell death [57].

### 3.3. The Microsomal Proteome of the ABA and H_2_O_2_ Responses Show Similarity

A total of 21 proteins were common among proteins significantly changing in response to ABA and H_2_O_2_ treatments. Ten of these proteins have been linked in RNA biology [39]. Changes in abundance for most of these proteins were similar (Table 3). Expression of six of these proteins increased in abundance at both 5 and 20 min post-treatment, while six decreased after either ABA or H_2_O_2_ treatment (Table 3). The other nine proteins showed dissimilar regulation patterns between the two treatments.

Three proteins, ubiquinol-cytochrome C reductase hinge (UQCRH, AT1G15120), D-3-phosphoglycerate dehydrogenase (AT1G17745) and NADH:cytochrome B5 reductase 1 (Cyt*b*5R, AT5G17770), belonging to the molecular function category ‘oxidoreductase activity’ (Table S2), showed differential accumulation in response to either ABA or H_2_O_2_ (Table 3). Oxidoreductase-related proteins have been shown to control responses to calcium homeostasis in organelles such as endoplasmic reticulum [58]. Besides, amongst the identified ABA and H_2_O_2_ –responsive proteins, UQCRH was one of the proteins increasing in abundance the most at both time points. It is a component of the mitochondrial respiratory chain complex III. Cyt*b*5R is an integral membrane-bound flavoprotein that is localised mainly in the endoplasmic reticulum and the outer mitochondrial membrane [59]. It is an electron donor for Cyt*b*5, a membrane-haeme-containing protein [60]. Non-cytotoxic concentration of H_2_O_2_ (24 μM) has been shown to induce a significant up-regulation of Cyt*b*5R, suggesting a redox regulation [61]. However, in this study, we observed that a higher concentration of H_2_O_2_ (50 μM) can cause a decrease of Cyt*b*5R abundance as an early response, and an increase at 20 min. This may suggest concentration-dependent redox regulation by Cyt*b*5R in response H_2_O_2_ as well as ABA.

Within the common proteins set, the most represented functional category was the cell structure, comprising of four tubulins and one actin. Tubulin/FtsZ protein (AT4G14960), an α-tubulin isoform that is required for right handed helical growth, is part of the tubulin complex or structural constituent of the cytoskeleton [62]. Tubulins showed differential expression in response to salt stress, for example, abundance of tubulin β-chain 2 (AT5G62690) decreased after salt treatment [63]. Tubulin has also been shown to regulate low nitrate-induced anthocyanin biosynthesis and plant nitrate response regulatory network in collaboration with other signalling pathways including ABA and H_2_O_2_ [64]. The four tubulins and actin 7 identified in the current study were all down-regulated in response to ABA and H_2_O_2_, and showed reduction in transcriptional level during senescence suggesting that the cytoskeletal organization-related proteins are affected by oxidative stress. Further, the cytoskeleton has been shown to be essential for mitochondrial morphology, movement and immobilization. The actin cytoskeleton, for example, is involved in the immobilization of mitochondria at the cortex in cultured tobacco cells, and the cytoskeleton may be critical for retaining mitochondria at sites of high ATP demand (for review see [65]).

**Figure 2:**
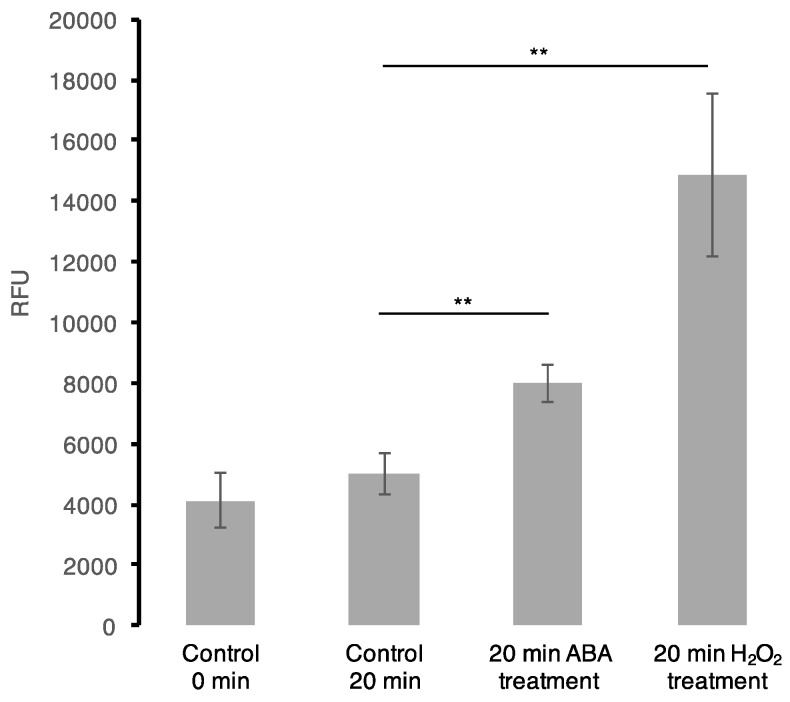
Reactive oxygen species (ROS) assay. OxiSelect™ Intracellular ROS assay kit (Cell Biolabs, Inc., San Diego, CA, USA) was used in the in vivo oxidation experiments using cultured Arabidopsis (Col-0) cells according to the assay protocol provided by the manufacturer. Each bar represents data from four biological replicates (*n* = 4) with a calculated standard error. Treatment of cells with ABA at the final concentration of 50 μM induces a statistically significant increase in H_2_O_2_ production, *p* < 0.001 (represented by two asterisks (**) symbols) using a two-sample *t*-test.

To further examine the mutual effect of ABA and H_2_O_2_, in particular to shed light on the common protein cohort whether the ABA effect is a result of the H_2_O_2_ signalling initiated via ABA pathway, we performed an ABA assay following H_2_O_2_ treatment of Arabidopsis cell suspension cultures and vise-versa. We noted no significant change in ABA quantity at 5 and 20 min post-H_2_O_2_ treatment. This may be attributed to the short treatment times aimed at observing early stimuli responses, or potentially from the low concentration of H_2_O_2_ used. Previously, it has been revealed that treating Arabidopsis guard cells with 100 μM H_2_O_2_ for 25 and 30 min induced an ABA-mediated nitric oxide production, which in turn is dependent on ABA-induced H_2_O_2_ synthesis [66]. Furthermore, a ROS assay was performed after treating Arabidopsis cell suspension cultures with 50 μM ABA. Here, we observed an increase in ROS accumulation at 20 min when compared with the controls (collected at 0 and 20 min) (Figure 2). This observation is not new as previously, ABA has been shown to induce H_2_O_2_ production through NADPH oxidase [67] and in guard cells of *Vicia faba*, ABA was also observed to generate H_2_O_2_ [68]. Additionally, in Arabidopsis, ROS accumulation was noted to occur upon ABA-induced stomatal closure [69]. Therefore, this suggests that ABA directly and/or indirectly induce ROS production in the cells that may lead to the observed common regulation of downstream processes with H_2_O_2_.

## 4. Conclusions

Shotgun proteomics approaches can distinguish common and specific responses linked to ABA or H_2_O_2_ treatments. The analysed proteomics data also revealed over 30 microsomal proteins associated with RNA interaction [70]. Furthermore, amongst the common set of proteins, we detected three proteins, namely aconitase 3, UQCRH, and inner mitochondrial membrane translocase 13, to be the most responsive and potentially as promising signatures for both ABA and H_2_O_2_ although they require further investigation to determine their mode of action. Besides, the fact that most of the common proteins changed in abundance in a similar manner is consistent with ABA-induced ROS production. Finally, we also show that a significant protein subset responds specifically to either ABA or H_2_O_2_, suggesting that these molecules also modulate independent complex intracellular responses.

## Figures and Tables

**Table 1:** Proteins responsive to H_2_O_2_ treatment.

Accession Number	Protein Name	ANOVA (*p*-Value)	FC 5 min	FC 20 min
**1. Metabolism**				
AT1G17745	D-3-phosphoglycerate dehydrogenase 2	0.0089	0.1	0.1
AT5G26780	Serine hydroxymethyltransferase 2	0.036	2.1	13
AT3G61440	Cysteine synthase C1	0.00074	ns	5
AT4G14880	O-acetylserine (thiol) lyase isoform A1	0.015	0.1	7.9
AT5G23300	Pyrimidine d	0.013	0.2	ns
AT3G09820	Adenosine kinase 1	0.0042	0.1	14.0
AT5G17770	NADH:cytochrome B5 reductase 1	0.017	0.4	2.5
AT1G74790	Catalytics	0.045	5.7	4.6
AT4G23850	AMP-dep. synthetase and ligase protein	0.017	0.5	ns
AT1G65290	Mitochondrial acyl carrier protein 2	0.044	2.9	ns
AT3G47930	L-galactono-1,4-lactone dehydrogenase	0.019	0.2	ns
**2. Energy**				
AT1G79550	Phosphoglycerate kinase	0.0069	0.2	2.0
AT1G24180	Pyruvate dehydrogenase E1 comp. α-2	0.014	0.1	0.2
AT3G48000	Aldehyde dehydrogenase 2B4	0.022	4.3	7.1
AT3G60750	Transketolase	0.0087	0.5	4.5
AT2G05710	Aconitase 3	0.0002	2.5	12.0
AT2G20420	ATP citrate lyase	0.022	ns	5.0
AT5G08300	Succinyl-CoA ligase, alpha subunit	0.006	2.4	2.5
AT2G44350	Citrate synthase family protein	0.026	ns	5.9
AT1G65930	Cyt. NADP^+^-dep. isocitrate dehydrogenase	0.033	0.1	2.9
AT1G15120	Ubiquinol-cytochrome C reductase hinge	0.016	19	20
AT3G03100	NADH:ubiquinone oxidoreductase,17.2kDa	0.0029	0.5	ns
AT5G13430	Ubiquinol-cytochrome C reductase, Fe-S	0.02	0.2	ns
AT3G14610	Cytochrome P450, 72A, polypeptide 7	0.0026	0.1	5.4
**3. Cell growth/division**				
AT5G52240	Membrane steroid binding protein 1	0.02	0.1	7.5
AT1G10930	DNA helicase (RECQl4A)	0.0036	4.6	0.1
AT3G44310	Nitrilase 1	0.023	2.9	ns
**4. Transcription**				
AT1G14850	Nucleoporin 155	0.00094	ns	0.3
**5. Protein synthesis**				
AT1G01100	60S acidic ribosomal	0.022	ns	0.4
AT3G48930	Nucleic acid-binding, OB-fold-like protein	0.024	2.1	ns
AT3G10090	Nucleic acid-binding, OB-fold-like protein	0.027	ns	0.3
AT3G09200	Ribosomal protein L10	0.012	2.9	ns
AT3G04400	Ribosomal protein L14p/L23e	0.0095	3.8	3.0
AT1G23290	Ribosomal protein L18e/L15	0.017	ns	0.5
AT4G02230	Ribosomal protein L19e	0.0089	ns	0.3
AT2G44120	Ribosomal protein L30/L7	0.027	ns	0.5
AT5G56710	Ribosomal protein L31e	0.013	4.6	ns
AT3G45030	Ribosomal protein S10p/S20e	0.03	ns	2.2
AT5G02960	Ribosomal protein S12/S23	0.016	2.3	ns
AT3G60245	Zinc-binding ribosomal protein	0.036	2.4	ns
**6. Protein destination and storage**				
AT1G14980	Chaperonin 10	0.022	ns	2.9
AT3G02530	Chaperonin 60 family protein	0.045	2.2	0.4
AT3G03960	Chaperonin 60 family protein	0.041	2.1	ns
AT5G56030	Heat shock protein 81-2	0.012	0.4	ns
AT3G52140	Tetratricopeptide repeat-containing protein	0.04	5.3	ns
**7. Transporters**				
AT4G38580	Farnesylated protein 6	0.017	0.4	ns
AT3G15660	Glutaredoxin 4	0.011	ns	6.0
AT1G27950	Glycosylphosphatidylinositol-anchored lipid protein transfer 1	0.027	16.0	44.0
AT1G07670	Endomembrane-type CA-ATPase 4	0.0026	0.1	5.0
AT4G27500	Proton pump interactor 1	0.006	ns	2.4
AT4G39080	Vacuolar proton ATPase A3	0.0041	0.5	ns
AT3G58730	Vacuolar proton pump D subunit	0.044	0.4	ns
AT1G15500	TLC ATP/ADP transporter	0.0099	0.3	ns
AT4G28390	ADP/ATP carrier 3	0.014	0.1	ns
AT5G60460	Preprotein translocase Sec, Sec61-β subunit	0.0081	4.7	4
AT3G51890	Clathrin light chain protein	0.019	ns	0.5
AT3G08530	Clathrin, heavy chain	0.0021	0.5	0.2
AT3G11130	Clathrin, heavy chain	0.00044	0.5	0.4
AT5G19760	Mitochondrial substrate carrier protein	0.038	ns	2.2
AT5G40810	Cytochrome C1	0.021	0.3	ns
**8. Intracellular traffic**				
AT3G49560	Mit. import inner membrane translocase Tim17	0.0031	0.1	ns
AT1G61570	Mit. import inner membrane translocase 13	0.0044	17	23
AT1G27390	Translocase outer membrane 20-2	0.0015	0.1	2.1
AT3G60600	Vesicle associated protein	0.051	ns	3
AT4G34660	SH3 domain-containing protein	0.039	4.7	ns
**9. Cell structure**				
AT5G09810	Actin 7	0.0038	0.5	ns
AT5G19770	Tubulin alpha-3	0.016	0.1	ns
AT5G62690	Tubulin beta chain 2	0.006	0.4	0.4
AT2G29550	Tubulin beta-7 chain	0.0034	0.3	0.4
AT4G14960	Tubulin/FtsZ family protein	0.036	0.4	0.5
AT3G08950	Electron transport SCO1/SenC protein	0.045	0.1	4.3
**10. Signal transduction**				
AT3G14840	LRR transmembrane protein kinase	0.025	ns	11
AT5G59840	Ras-related small GTP-binding protein	0.0084	0.1	2.7
AT3G51800	Metallopeptidase M24	0.0015	5.1	ns
**11. Disease/Defence**				
AT3G57280	Transmembrane proteins 14C	0.014	0.1	ns
AT3G12500	Basic chitinase	0.035	ns	2.1
AT3G32980	Peroxidase superfamily protein	0.012	ns	3
AT4G36430	Peroxidase superfamily protein	0.015	0.1	ns
**12. Unclassified**				
AT2G40765	Unknown protein	0.019	2	ns
AT2G46540	Unknown protein	0.033	ns	3.5
AT4G12590	Transmembrane protein, DUF106	0.033	0.3	ns
AT1G08480	Unknown protein	0.048	0.2	0.4
AT3G20370	TRAF-like family protein	0.0058	0.2	3
AT3G49720	Unknown protein	0.016	0.1	0.3
AT4G24330	DUF1682, unknown protein	0.0077	0.1	2.2
AT2G33585	Unknown protein	0.019	0.1	13

Notes: no significant change against the mock treated samples; dep.: dependent; comp.: component; cyt.: cytosolic; ANOVA: Analysis Of Variance.

**Table 2:** Proteins responsive to abscisic acid (ABA) treatment.

Accession Number	Protein Name	ANOVA (*p*-Value)	FC 5 min	FC 20 min
**1. Metabolism**				
AT2G26400	Acireductone dioxygenase 3	0.035	3.9	6.8
AT4G34200	D-3-phosphoglycerate dehydrogenase 1	0.017	0.5	ns
AT1G17745	D-3-phosphoglycerate dehydrogenase 2	0.029	0.1	0.2
AT4G13940	S-adenosyl-L-homocysteine hydrolase	0.028	2.5	2.0
AT3G17820	Glutamine synthetase 1.3	0.023	3.7	3.5
AT5G17770	NADH cytochrome B5 reductase 1	0.046	2.3	2.3
AT3G15730	Phospholipase D α 1	0.014	3.2	3.8
**2. Energy**				
AT2G01140	Aldolase superfamily protein	0.0075	5.0	5.1
AT5G43940	GroES-like zinc-binding dehydrogenase	0.014	4.5	5.2
AT4G34870	Rotamase cyclophilin 5	0.0015	3.1	2.7
AT2G05710	Aconitase 3	0.035	8.0	9.9
AT1G15120	Ubiquinol-cytochrome C reductase hinge	0.041	15.0	11.0
AT2G33220	GRIM-19 protein	0.017	2.9	7.1
**3. Cell growth/division**				
AT5G43070	WPP domain protein 1	0.042	0.4	0.2
**4. Transcription**				
AT2G18740	Small nuclear ribonucleoprotein	0.02	7.7	4.3
AT5G40480	Embryo defective 3012	0.0084	5.6	7.9
AT1G14850	Nucleoporin 155	0.0058	0.1	0.2
**5. Protein synthesis**				
AT1G08360	Ribosomal protein L1p/L10e	0.037	3.1	ns
AT4G10450	Ribosomal protein L6	0.00092	12.0	>0.1
AT5G08180	Ribosomal protein L7Ae	0.022	11.0	8.7
AT3G04400	Ribosomal protein L14p	0.016	3.5	2.6
AT3G14600	Ribosomal protein L18ae	0.033	3.4	3.3
AT3G45030	Ribosomal protein S10p/S20e	0.0041	2.3	ns
AT5G47930	Zinc-binding ribosomal protein	0.027	8.6	7.0
AT4G00810	60S acidic ribosomal protein family	0.042	0.4	0.4
AT1G01100	60S acidic ribosomal protein family	0.045	0.4	0.4
AT5G47700	60S acidic ribosomal protein family	0.0085	0.3	0.4
**6. Protein destination and storage**				
AT1G14980	Chaperonin 10	0.0061	2.5	3.0
AT3G16420	PYK10-binding protein 1	0.017	0.3	0.5
AT5G56030	Heat shock protein 81-2	0.0038	0.4	0.5
AT1G03220	Eukaryotic aspartyl protease	0.0045	2.3	2.5
**7. Transporters**				
AT3G53420	Plasma membrane intrinsic protein 2A	0.031	0.3	ns
AT3G42050	Vacuolar ATP synthase subunit H	0.045	4.4	4.2
AT4G27500	Proton pump interactor 1	0.0048	ns	2.3
AT2G34250	SecY protein transport	0.011	2.5	3.9
AT3G08530	Clathrin, heavy chain	0.0019	0.2	0.4
AT3G11130	Clathrin, heavy chain	0.00054	0.3	0.4
**8. Intracellular traffic**				
AT2G29530	TIM10 zinc finger protein	0.021	6.0	5.0
AT1G61570	Mit. import inner membrane 13 translocase	0.042	19.0	11.0
**9. Cell structure**				
AT4G30270	Xyloglucan endotransglucosylase	0.045	ns	3.0
AT3G18780	Actin 2	0.016	0.3	ns
AT5G09810	Actin 7	0.00073	0.5	0.5
AT5G19770	Tubulin alpha-3	0.044	0.5	0.3
AT5G62690	Tubulin beta chain 2	0.003	0.3	0.4
AT2G29550	Tubulin beta-7 chain	0.0072	0.3	0.4
AT4G14960	Tubulin/FtsZ family protein	0.019	0.4	0.3
**10. Signal transduction**				
AT3G14840	LRR transmembrane protein kinase	0.014	8.3	14.0
AT3G51800	Metallopeptidase M24	0.027	3.4	ns
**11. Disease/Defence**				
AT1G78850	D-mannose binding lectin protein	0.0013	14.0	18.0
AT2G43610	Chitinase family protein	0.05	ns	0.3
AT4G38740	Rotamase CYP 1	0.02	2.6	ns
AT1G20620	Catalase 3	0.0064	3.5	2.1

Notes: no significant change against the mock treated samples.

**Table 3:** Comparison differential protein accumulation in response to ABA and H_2_O_2_.

Accession Number	Protein Name	H_2_O_2_ 5 min	H_2_O_2_ 20 min	ABA 5 min	ABA 20 min
**1. Metabolism**					
AT1G17745	D-3-phosphoglycerate dehydrogenase 2	0.1	0.1	0.1	0.2
AT5G17770	NADH:cytochrome B5 reductase 1	0.4	2.5	2.3	2.3
**2. Energy**					
AT2G05710	Aconitase 3	2.5	12	8	9.9
AT1G15120	Ubiquinol-cytochrome C reductase hinge	19	20	15	11
**3. Transcription**					
AT1G14850	Nucleoporin 155	ns	0.3	0.1	0.2
**4. Protein synthesis**					
AT3G04400	Ribosomal protein L14p/L23e	3.8	3	3.5	2.6
AT3G45030	Ribosomal protein S10p/S20e	ns	2.2	2.3	ns
AT1G01100	60S acidic ribosomal protein family	ns	0.4	0.4	0.4
**5. Protein destination and storage**					
AT1G14980	Chaperonin 10	ns	2.9	2.5	3
AT5G56030	Heat shock protein 81-2	0.4	ns	0.4	0.5
**6. Transporters**					
AT4G27500	Proton pump interactor 1	ns	2.4	ns	2.3
AT3G08530	Clathrin, heavy chain	0.5	0.2	0.2	0.4
AT3G11130	Clathrin, heavy chain	0.5	0.4	0.3	0.4
**7. Intracellular traffic**					
AT1G61570	Mit. import inner membrane 13 translocase	17	23	19	11
**8. Cell structure**					
AT5G09810	Actin 7	0.5	ns	0.5	0.5
AT4G14960	Tubulin/FtsZ family protein	0.4	0.5	0.4	0.3
AT5G62690	Tubulin beta chain 2	0.4	0.4	0.3	0.4
AT2G29550	Tubulin beta-7 chain	0.3	0.4	0.3	0.4
AT5G19770	Tubulin alpha-3	0.1	ns	0.5	0.3
**9. Signal transduction**					
AT3G51800	Metallopeptidase M24 family protein	5.1	ns	3.4	ns
AT3G14840	LRR transmembrane protein kinase	ns	11	8.3	14

## Supplementary Materials

The following are available online http://www.mdpi.com/2227-7382/5/3/22/s1, Table S2: H_2_O_2_ differentially expressed proteins and GO analysis, Table S3: ABA differentially expressed proteins and GO analysis.
